# Four cardiomyopathy patients with a heterozygous *DSG2* p.Arg119Ter variant

**DOI:** 10.1038/s41439-024-00304-w

**Published:** 2024-12-20

**Authors:** Takuya Sumida, Shou Ogawa, Shuichiro Higo, Yuki Kuramoto, Ryo Eto, Yoshihiko Ikeda, Congcong Sun, Junjun Li, Li Liu, Tomoka Tabata, Yoshihiro Asano, Mikio Shiba, Yasuhiro Akazawa, Daisuke Nakamura, Takafumi Oka, Tomohito Ohtani, Yasushi Sakata

**Affiliations:** 1 Faculty of Medicine, Osaka University, Suita, Osaka 565-0871 Japan; 2 Department of Cardiovascular Medicine, Osaka University Graduate School of Medicine, Suita, Osaka 565-0871 Japan; 3 Department of Pathology, National Cerebral and Cardiovascular Center, Suita, Osaka 564-8565 Japan; 4 Photonics Cell Evaluation Laboratory, Graduate School of Engineering, Osaka University, Suita, Osaka 565-0871 Japan; 5 Department of Genomic Medicine, National Cerebral and Cardiovascular Center, Suita, Osaka 564-8565 Japan; 6 Cardiovascular Division, Osaka Police Hospital, Osaka, 543-0035 Japan

**Keywords:** Cardiomyopathies, Disease genetics

## Abstract

*DSG2*, encoding desmoglein-2, is one of the causative genes of arrhythmogenic cardiomyopathy. We previously identified a homozygous *DSG2* p.Arg119Ter stop-gain variant in a patient with juvenile-onset cardiomyopathy and advanced biventricular heart failure. However, the pathological significance and prevalence of the heterozygous *DSG2* p.Arg119Ter variant remains uncertain. Here, we identified four unrelated patients with cardiomyopathy with heterozygous *DSG2* p.Arg119Ter variants among 808 patients with nonischemic cardiomyopathy; the allele frequency was 0.0037, which is more than 50-fold greater than that reported in the general Japanese population. These patients were clinically diagnosed with arrhythmogenic right ventricular cardiomyopathy (Pt-1), dilated cardiomyopathy (DCM) after ventricular septum defect closure surgery (Pt-2), DCM (Pt-3), and end-stage hypertrophic cardiomyopathy (Pt-4). The patients also exhibited reduced left ventricular contractile function and varying clinical courses. Genetic analysis identified additional possible causative variants, *DSG2* p.Arg292Cys in Pt-1 and *BAG3* p.His166SerfsTer6 in Pt-3. Immunohistochemical analysis of endomyocardial biopsy samples revealed that the expression of not only desmoglein-2 but also desmoplakin was markedly reduced. Transmission electron microscopy revealed pale and fragmented desmosomes and widened gaps between intercalated discs in the myocardium. A microforce test using human cardiomyocytes differentiated from induced pluripotent stem cells (iPSC-CMs) demonstrated reduced contractility in iPSC-CMs carrying a heterozygous truncating variant in *DSG2*. These data suggest that the *DSG2* p.Arg119Ter variant is concealed in patients with cardiomyopathy with heart failure, and desmosome impairment may be a latent exacerbating factor of contractile dysfunction and disease progression.

## DSG2 Variant Linked to Severe Cardiomyopathy Cases

This study explores the genetic factors behind certain heart diseases, focusing on a gene called *DSG2*. Variants in *DSG2* can lead to arrhythmogenic cardiomyopathy (AC), a condition causing heart rhythm problems and heart muscle dysfunction. Researchers found a specific *DSG2* variant, p.Arg119Ter, in four patients with nonischemic cardiomyopathy.The study involved genetic analysis of 808 patients, identifying five with *DSG2* variants. Researchers used techniques like whole-exome sequencing and immunohistochemical analysis. They found that the *DSG2* variant might contribute to heart failure by affecting desmosomes.The results suggest that the *DSG2* p.Arg119Ter variant is more common in East Asian populations and may worsen heart conditions when combined with other genetic or environmental factors. This research highlights the importance of genetic screening for better understanding and managing heart diseases. "This summary was initially drafted using artificial intelligence, then revised and fact-checked by the author."

## Introduction

Recent genetic studies using high-throughput sequencing technologies have identified various pathogenic variants and revealed an association between genetic factors and clinical phenotypes in patients with cardiomyopathies^1,2^. Desmosomal genes (*PKP2*, *JUP*, *DSP*, *DSC2*, and *DSG2*) encode the structural proteins of the desmosome, a dynamic junctional component that maintains the structural integrity of heart tissues; genetic variants in these desmosomal genes cause arrhythmogenic cardiomyopathy (AC) characterized by lethal arrhythmia and myocardial dysfunction, predominantly in the right ventricle^3–6^. Desmoglein-2, encoded by *DSG2*, is a desmosomal cadherin protein that is expressed mainly in heart tissue, and heterozygous missense or nonsense mutations in *DSG2* have been identified in patients with AC^7–10^. We previously identified a patient with juvenile-onset severe biventricular heart failure carrying a homozygous stop-gain variant of *DSG2* (NM_001943.5(NP_001934.2):c.355 C > T, p.Arg119Ter) and demonstrated that complete desmoglein-2 deficiency causes abnormal deposition of desmosome proteins and disruption of intercalated discs in cardiomyocytes^11^. Although the deleterious effect of the homozygous *DSG2* p.Arg119Ter variant is evident, the interpretation of the effects of the heterozygous *DSG2* p.Arg119Ter variant is conflicting (ClinVar database)^12^, and its pathological role remains uncertain.

In this study, we identified four unrelated cardiomyopathy patients with heterozygous *DSG2* p.Arg119Ter variants among 808 patients with nonischemic cardiomyopathy. The clinical diagnoses and clinical courses varied among these four patients. Genetic analysis identified additional possible causative variants, and immunohistochemical analysis of endomyocardial biopsy samples demonstrated reduced expression of not only desmoglein-2 but also desmoplakin. Transmission electron microscopy revealed pale and fragmented desmosomes and widened gaps between intercalated discs in the myocardium. These data suggest that the *DSG2* p.Arg119Ter variant may be concealed in patients with cardiomyopathy with heart failure, and desmosome impairment combined with genetic or environmental factors may promote contractile dysfunction.

## Materials and methods

### Genetic analysis

Genetic analysis was approved by the Ethics Committee of Osaka University (accession number: 680,684). All investigations conformed to the Ethical Guidelines for Medical and Health Research Involving Human Subjects in Japan. Informed consent was obtained from all patients. Genetic analyses were performed as previously described^13^ with slight modifications. Briefly, genomic DNA was extracted from peripheral blood, and whole-exome sequencing was performed. The sequencing results were searched for rare pathogenic variants of 57 known causative genes of cardiomyopathy^13^. Rare pathogenic variants were identified by applying the following criteria: (i) exonic or splice-site variants excluding exonic synonymous variants; (ii) variants included in the gene list; (iii) variants with a minor allele frequency <5%; (iv-1) variants listed as disease-causing or likely disease-causing mutations in the HGMD (2021)^14^ or as pathogenic variants or likely pathogenic variants in ClinVar (clinvar_20210501)^12^; (iv-2) nonsense, frameshift or splice-site variants; and (iv-3) homozygous variants with a minor allele frequency <0.5% or heterozygous variants with a minor allele frequency <0.005%. Variants meeting the criteria (i), (ii), (iii) and ((iv-1) or (iv-2) or (iv-3)) are listed in Table 2. The targeted genomic regions were amplified via PCR (KOD Fx Neo, TOYOBO), purified, and analyzed via direct Sanger sequencing or cloning (pCR bluntII-TOPO vector, Thermo) via PCR primers (Supplementary Table 1).

### Immunohistochemical analysis and transmission electron microscopy (EM) analysis

One of the myocardial samples collected was subjected to EM evaluation; the remaining samples were subjected to light microscopy examination. Masson’s trichrome staining and immunohistochemistry were performed on 2-µm-thick formalin-fixed and paraffin-embedded tissue sections from the right ventricular wall. We used a fully automated immunohistochemical staining system, Bond-III (Leica Microsystems). Briefly, the samples were deparaffinized, and antigens were retrieved from the instrument. All the slides were incubated with primary antibodies against desmoglein-2 (diluted 1:10; PROGEN 651119), desmoplakin (diluted 1:10; PROGEN 65146), plakoglobin (diluted 1:10; PROGEN 65105), and plakophilin-2 (diluted 1:10; PROGEN 651101). Antibody binding was visualized via the avidin-biotin complex method according to the manufacturer’s instructions (Vectastain ABC; Vector). Microstructural abnormalities were assessed by sequential EM. Endomyocardial biopsy samples were fixed in 2.5% glutaraldehyde and post-fixed in 1% osmium tetroxide. The samples were dehydrated in ethanol and embedded in Epok 812 (Ernest F. Fullam, Inc., Latham, NY, USA). Ultrathin sections were cut using an ultramicrotome, stained with lead citrate and uranyl acetate, and examined under an electron microscope (HT7650; Hitachi Ltd., Ibaraki, Japan). Induced pluripotent stem cells (iPSCs) generated from a patient carrying the homozygous *DSG2* p.Arg119Ter variant were differentiated into cardiomyocytes (iPSC-CMs) and fixed for EM evaluation as previously described^6^.

### Microforce test using self-organized tissue rings

Self-organized tissue rings (SOTRs)^15^ were generated from iPSC-CMs carrying a heterozygous frameshift variant (c.297dupT, p.Gly100TrpfsTer105) in *DSG2*^11^ and from normal iPSC-CMs without a pathogenic variant in *DSG2* as a control (iPSC-CMs were generated from a patient with ARVC in which the genetic variant in *PKP2* is normally corrected by genome editing^6^). The active force of the SOTRs was measured with a micron-scale mechanical testing system (MicroTester G2; CellScale Biomaterials Testing) as previously described^16^.

## Results

### Prevalence of the *DSG2* (c.355 C > T, p.Arg119Ter) variant in nonischemic cardiomyopathies

*DSG2* (c.355 C > T, p.Arg119Ter) is an extremely rare variant in the gnomAD database of exome and genome sequences worldwide, with a minor allele frequency (MAF) of 0.000009297 (9/44,826 in the East Asian population, 6/1,179,852 in the European population (non-Finnish), and none in other cohorts)^17^. Importantly, the MAFs of *DSG2* (c.355 C > T, p.Arg119Ter) in the East Asian population (0.0002008) and in the Japanese cohort in the Tohoku Medical Megabank (0.000064 (7/108,574)) (jMorp database)^18^ were approximately 21.6-fold and 6.9-fold higher, respectively, than those in the total gnomAD database (Table 1). We screened 808 patients diagnosed with nonischemic cardiomyopathy at Osaka University Hospital from 2011 to 2023 via exome sequence analysis and identified five patients with *DSG2* (c.355 C > T, p.Arg119Ter) variants, including one patient with a previously reported homozygous variant^11^ and four patients with heterozygous variants. The MAF of *DSG2* (c.355 C > T, p.Arg119Ter) was 0.003712871, which was more than 50-fold greater than that in the general Japanese population. These data suggest that the *DSG2* (c.355 C > T, p.Arg119Ter) variant is more common in East Asian populations than in other cohorts and is concentrated in patients with nonischemic cardiomyopathy (Table 1).

**Table 1:** Allele frequency of *DSG2* (c.355 C > T, p.Arg119Ter) variant in the large cohort studies and in 808 non-ischemic cardiomyopathy patients in Osaka University Hospital.

Dataset	Genetic ancestry group	Allele count	Allele number	Allele frequency
gnomAD^15^	East Asian	9	44,826	0.0002008
	European (non-Finnish)	6	1,179,852	0.000005085
	Others	0	388,792	0
	Total	15	1,613,470	0.000009297
ToMMo 54KJPN^16^		7	108,574	0.000064472
808 non-ischemic cardiomyopathy patients Osaka University Hospital		6 1 homozygous 4 heterozygous	1616	0.003712871

### Clinical courses of cardiomyopathy patients with heterozygous *DSG2* (c.355 C > T, p.Arg119Ter) variants

**Fig. 1:**
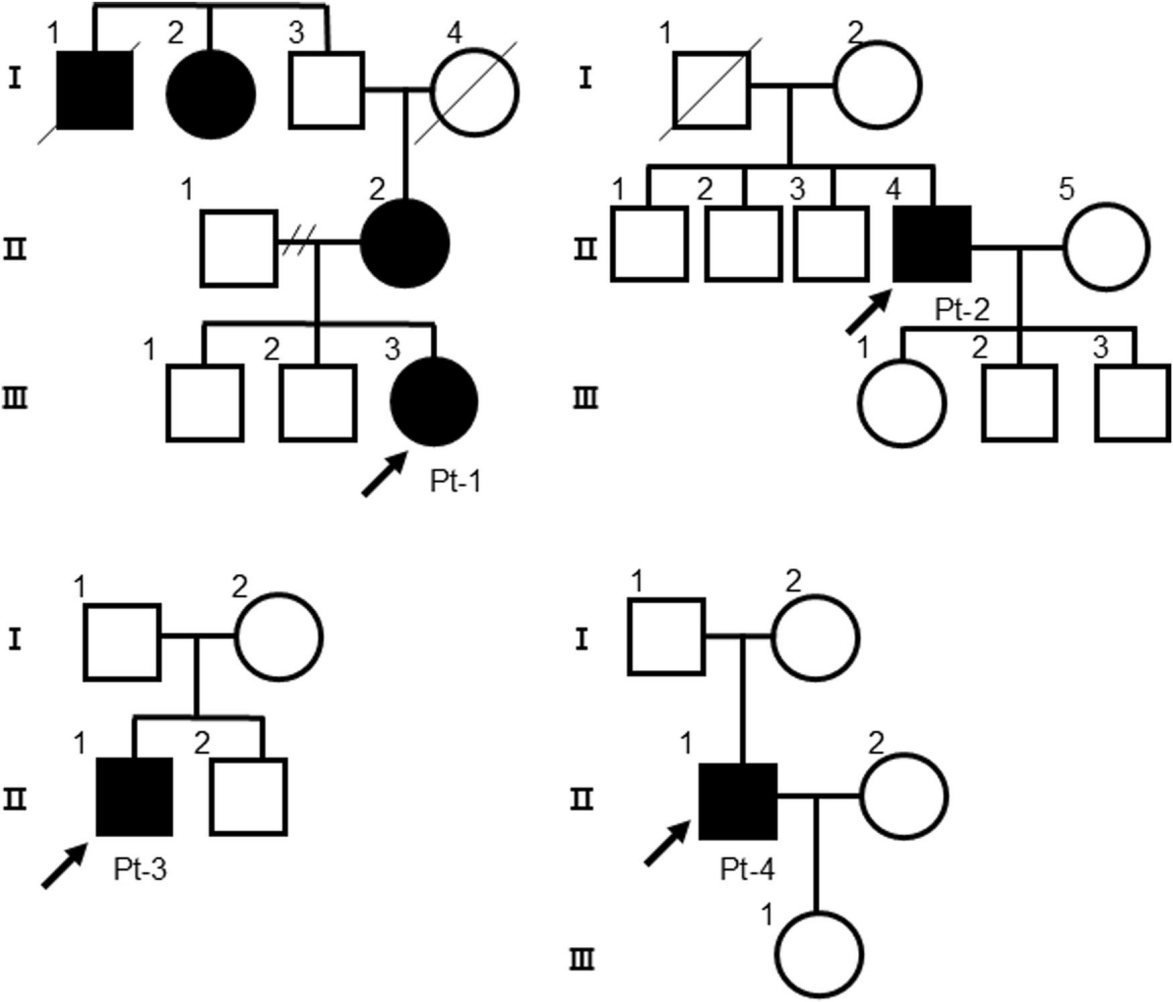
Family pedigree charts of patients with the *DSG2* (c.355 C > T, p.Arg119Ter) variant. The black arrows indicate Pt-1 (III-3), Pt-2 (II-4), Pt-3 (II-1), and Pt-4 (II-1). A family history of cardiovascular diseases or abnormal findings on cardiovascular examination (indicated as black squares or circles) was observed in Pt-1 (I-1: sudden death at 73 y; I-2: pacemaker implantation; II-2: enlarged heart on chest X-ray) but not in Pt-2, 3, or 4.

**Fig. 2:**
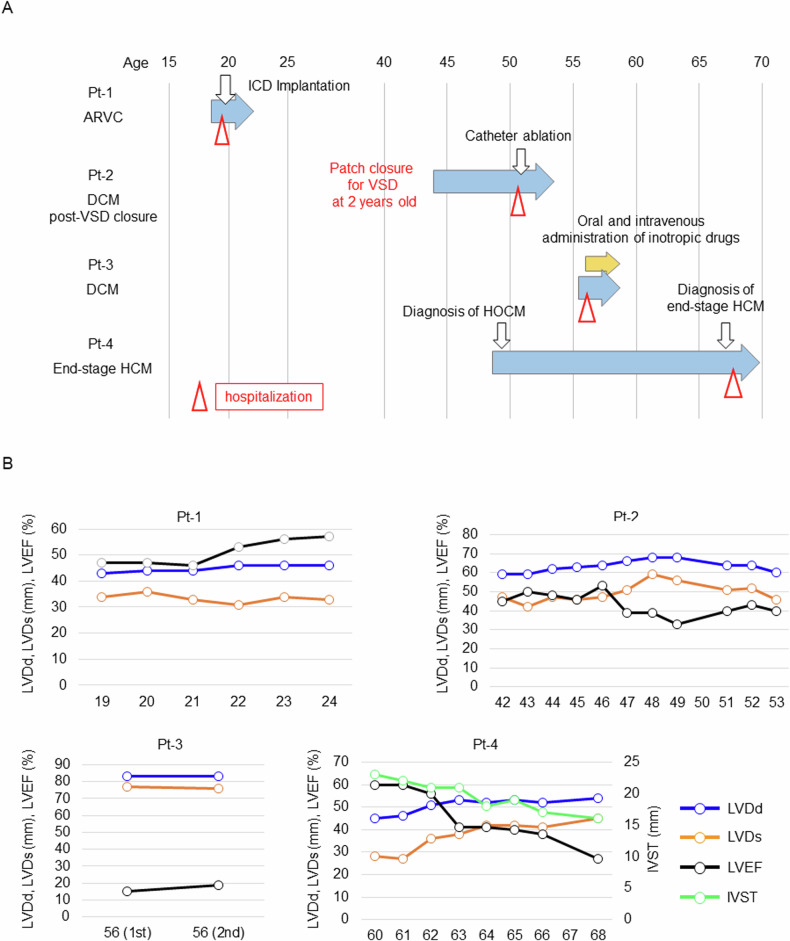
Clinical courses and time courses of echocardiographic parameters of the four patients. **A** Clinical courses of the four patients. ICD implantable cardioverter-defibrillator. VSD ventricular septal defect, HOCM hypertrophic obstructive cardiomyopathy, HCM hypertrophic cardiomyopathy. **B** Echocardiographic parameters of the four patients during the clinical course collected from their medical records. LVDd left ventricular end-diastolic diameter, LVDs left ventricular end-systolic diameter, IVST interventricular septum thickness, LVEF left ventricular ejection fraction. The X-axis indicates the age of each patient at echocardiographic evaluation.

The patient with a homozygous *DSG2* (c.355 C > T, p.Arg119Ter) variant presented with juvenile-onset severe biventricular heart failure^11^, whereas patients with heterozygous variants presented various diagnoses and clinical courses. These four patients (patient (Pt)-1, 2, 3, and 4) were identified within unrelated families, and only Pt-1 had a family history of cardiovascular disease or abnormal findings on cardiovascular examination (Fig. 1). Figure 2 shows the clinical courses and time courses of the echocardiographic parameters of these patients. Pt-1 (a 19-year-old woman) was aware of congestion at 17 years of age and was diagnosed with arrhythmogenic right ventricular cardiomyopathy (ARVC) according to the TASC Force Criteria in 2010 at 19 years of age^19^. The mother of the proband (II-2) carried a heterozygous *DSG2* (c.355 C > T, p.Arg119Ter) variant and presented with an enlarged heart on a chest X-ray, but she was not diagnosed with ARVC. An echocardiogram revealed a left ventricular ejection fraction (LVEF) of 47–57% (normal range: 66 ± 5% in women^20^), suggesting not only right ventricular but also left ventricular contractile dysfunction. Cardiac MRI revealed right ventricular dilatation and reduced contraction of both the right and left ventricles but did not detect fatty degeneration. Owing to the diagnosis of ARVC and frequent premature ventricular contractions, Pt-1 received an implantable cardioverter-defibrillator (ICD). Pt-2 (a 52-year-old man) underwent patch closure for a ventricular septum defect (VSD) at 2 years of age via open heart surgery and did not experience any cardiac symptoms until his thirties. From the age of 40 years, the left ventricular diastolic and systolic diameters (LVDd/LVDs) gradually increased, the LVEF gradually decreased (LVDd/Ds: 64/51 mm, LVEF: 40% at 50 years of age), and nonsustained ventricular tachycardia (NSVT) was detected. Pt-2 was diagnosed with dilated cardiomyopathy (DCM) at 52 years of age and underwent catheter ablation for symptomatic atrial fibrillation and atrial flutter. Pt-3 (a 56-year-old man) developed fatigue and shortness of breath for the first time and was subsequently diagnosed with DCM. Echocardiographic evaluation revealed a markedly enlarged left ventricle and severe left ventricular dysfunction (LVDd/s: 83/77 mm, LVEF: 15%). Oral and intravenous administration of inotropic drugs was required because of severe advanced heart failure. Pt-4 (a 68-year-old man) was diagnosed with hypertrophic obstructive cardiomyopathy (HOCM) via echocardiogram in his 40 s. From the age of 60 years, the LVDd/LVDs gradually increased, and the LVEF and intraventricular septum thickness (IVST) gradually decreased. Pt-4 was diagnosed with end-stage hypertrophic cardiomyopathy (HCM) at 68 years of age.

### Pathogenic variants identified in the four cardiomyopathy patients with *DSG2* (c.355 C > T, p.Arg119Ter)

The pathogenic variants identified in these patients after filtering the results of whole-exome sequencing analysis followed by Sanger sequencing analysis are shown in Table 2 and Figs. S1, S2. A variant list of all the data used for the filtering procedure is shown in Supplementary Table 2. In Pt-1, the *DSG2* (c.874 C > T, p.Arg292Cys) variant was identified, and Sanger sequencing after PCR cloning confirmed that *DSG2* p.Arg119Ter and p.Arg292Cys were compound heterozygous (Fig. S1). In Pt-2, no rare pathogenic variants other than the *DSG2* p.Arg119Ter variant were identified. In Pt-3, a heterozygous frameshift variant (c.486_487insCCAGCCT, p.His166SerfsTer6) was identified in *BAG3* (NM_004281.4(NP_004272.2)), encoding BLC2-associated athanogene 3 located on the sarcomere Z-disc, which is a causative gene of DCM^21^ (Fig. S2). In Pt-4, no pathogenic variants were identified in the established causal genes associated with sarcomere function (*MYH7*, *MYBPC3*, *TNNT2*, *TNNI3*, *TPM1*, *ACTC1*, *MYL2*, *MYL3*, and *CSRP3*)^22^, although the clinical diagnosis of Pt-4 was HOCM. Five rare variants were identified after the filtering procedure in Pt-1 and Pt-4 (*EYA4* p.Pro170Thr, *TTN* p.Glu22134Lys, and *VCL* p.Pro291Gln in Pt-1 and *RYR2* p.Val1810Leu and *TTN* p.Arg22007Cys in Pt-4); however, based on previous studies and genetic databases, it was not evident whether these variants were related to the development of ARVC in Pt-1 or HCM in Pt-4.

**Table 2:** Pathogenic genetic variants identified in the four patients with *DSG2* (c.355 C > T, p.Arg119Ter).

Patient	Diagnosis	Position (hg38)	Gene	Zygosity	Base change	Amino acid change	Variant type
Pt-1	ARVC	18:31520941	*DSG2*	Heterozygous	c.355 C > T	p.Arg119Ter	stopgain
		18:31524748	*DSG2*	Heterozygous	c.874 C > T	p.Arg292Cys	nonsynonymous SNV
		6:133462710	*EYA4*	Heterozygous	c.508 C > A	p.Pro170Thr	nonsynonymous SNV
		2:178548031	*TTN*	Heterozygous	c.66400 G > A	p.Glu22134Lys	nonsynonymous SNV
		10:74082542	*VCL*	Heterozygous	c.872 C > A	p.Pro291Gln	nonsynonymous SNV
Pt-2	DCM post VSD closure	18:31520941	*DSG2*	Heterozygous	c.355 C > T	p.Arg119Ter	stopgain
Pt-3	DCM	18:31520941	*DSG2*	Heterozygous	c.355 C > T	p.Arg119Ter	stopgain
		10:119670156	*BAG3*	Heterozygous	c.486_487insCCAGCCT	p.His166SerfsTer6	frameshift insertion
Pt-4	End-stage HCM	18:31520941	*DSG2*	Heterozygous	c.355 C > T	p.Arg119Ter	stopgain
		1:237614556	*RYR2*	Heterozygous	c.5428 G > C	p.Val1810Leu	nonsynonymous SNV
		2:178548412	*TTN*	Heterozygous	c.66019 C > T	p.Arg22007Cys	nonsynonymous SNV

### Histopathological evaluation of the patients’ myocardium

**Fig. 3:**
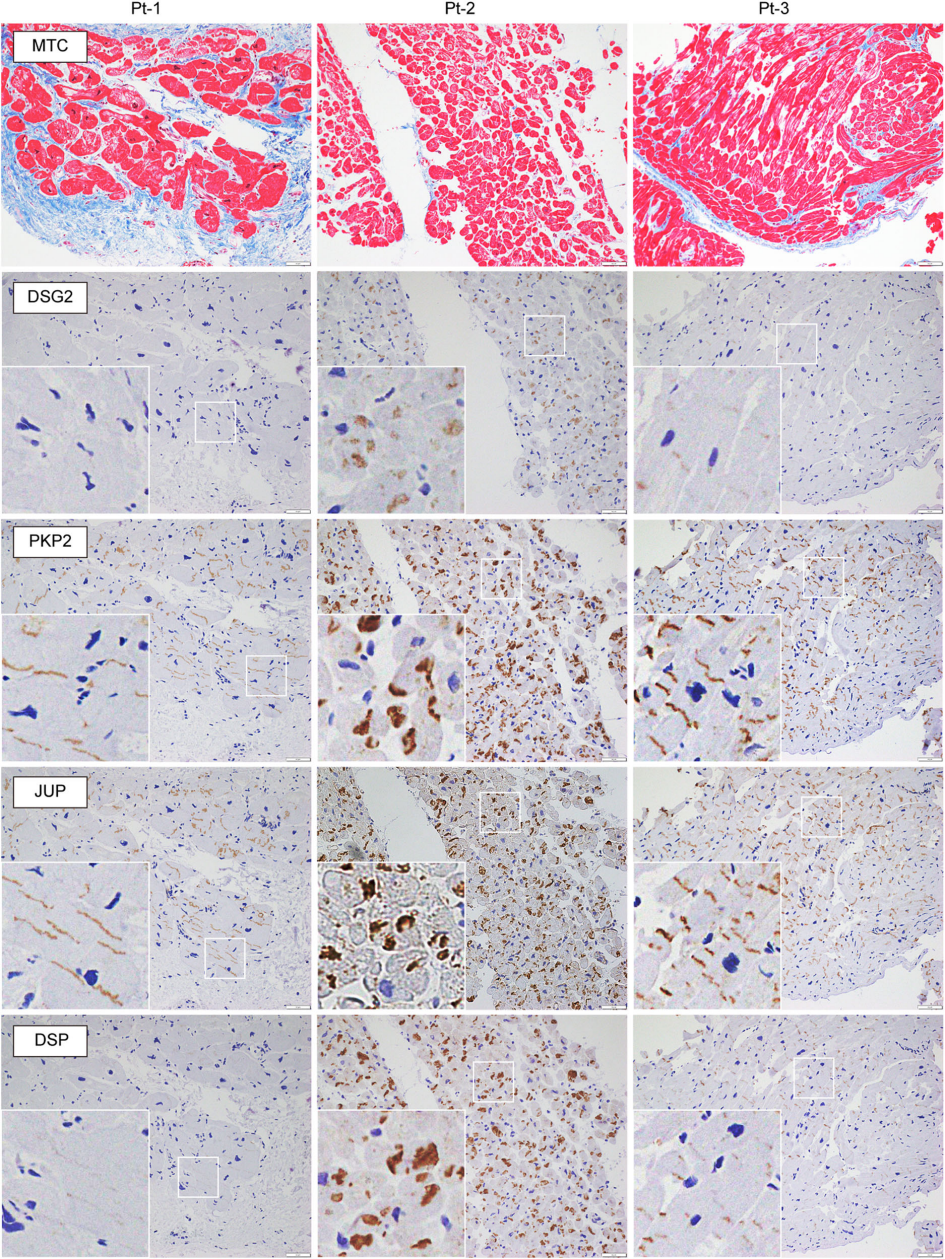
Histopathological evaluation of myocardial samples of the patients. Masson’s trichrome (MTC) staining and immunohistochemical analysis of right ventricular myocardial biopsy samples obtained from Pt-1, Pt-2, and Pt-3. The white squares are enlarged for each sample. Scale bars: 50 μm.

**Fig. 4:**
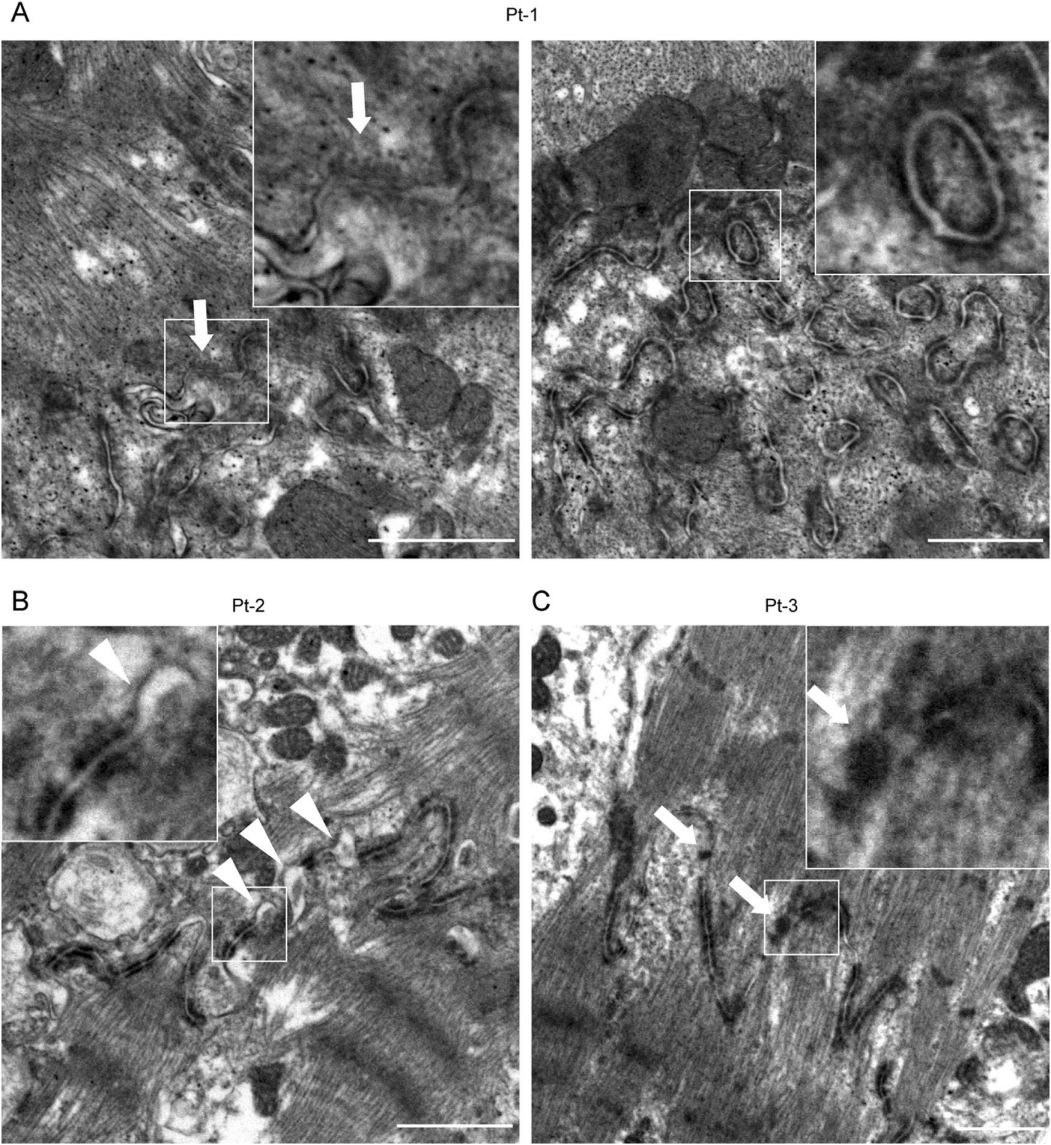
Transmission electron microscopy analysis of myocardial samples of the patients. Transmission electron microscopy images of the right ventricular myocardium. The white squares are enlarged in each panel. **A** Abnormal pale desmosomes (arrow, left panel) and fragmented aggregated desmosomes (right panel) were observed in Pt-1. **B** Widened gaps in the intercalated disc structures (arrowheads) were observed in Pt-2. **C** Abnormal pale desmosomes (arrows) were observed in Pt-3. Scale bars: 1 μm.

We previously demonstrated that the complete loss of desmoglein-2 due to the homozygous *DSG2* p.Arg119Ter variant causes abnormal deposition of desmosome proteins and disruption of intercalated discs in cardiomyocytes, and iPSC-CMs generated from patients recapitulate reduced contractility, abnormal excitation, and aberrant myocardial fiber structures^11^. We confirmed the pathological findings in desmosomes from both the LV myocardium and iPSC-CMs generated from the patient with the homozygous *DSG2* p.Arg119Ter variant by transmission electron microscopy analysis (Fig. S3). To clarify how the heterozygous *DSG2* p.Arg119Ter variant affects the expression of desmosome proteins, serial sections of myocardium samples obtained by right ventricular myocardial biopsy on Pts 1, 2, and 3 were examined by immunostaining. Pt-4 did not undergo myocardial biopsy. Masson’s trichrome (MTC) staining revealed massive fibrosis in Pt-1 and modest fibrosis in Pt-2 and Pt-3 (Fig. 3). Desmoglein-2 reactivity was diminished in Pt-1 as evaluated by immunostaining, suggesting that the combination of these variants (Arg119Ter and Arg292Cys) severely affected protein expression. Desmoglein-2 reactivity decreased in Pt-3 but was preserved in Pt-2. The reactivities of plakophilin-2 and plakoglobin were preserved in Pts 1, 2 and 3. Notably, desmoplakin reactivity was significantly decreased in Pt-1 and Pt-3. Although the expression of desmosome proteins was preserved in Pt-2, their distribution in the intercalated discs exhibited abnormal deposition rather than a normal peripheral distribution. The myocardial samples were further evaluated via transmission electron microscopy. Abnormal pale junctions characterized by repeating couplings, elongated desmosomes, and widened gaps in intercalated discs, typically observed in ARVC patients^23^, were detected in the myocardium of Pt-1, Pt-2, and Pt-3 (Figs. 4A–C). Notably, fragmented and aggregated desmosomes were detected in the myocardium of Pt-1 (Fig. 4A). Similar findings were observed in a patient with biventricular heart failure due to complete loss of desmoglein-2 caused by the homozygous *DSG2* p.Arg119Ter variant^11^.

### Reduced contractility in iPSC-CMs carrying a heterozygous truncating variant in *DSG2*

**Fig. 5:**
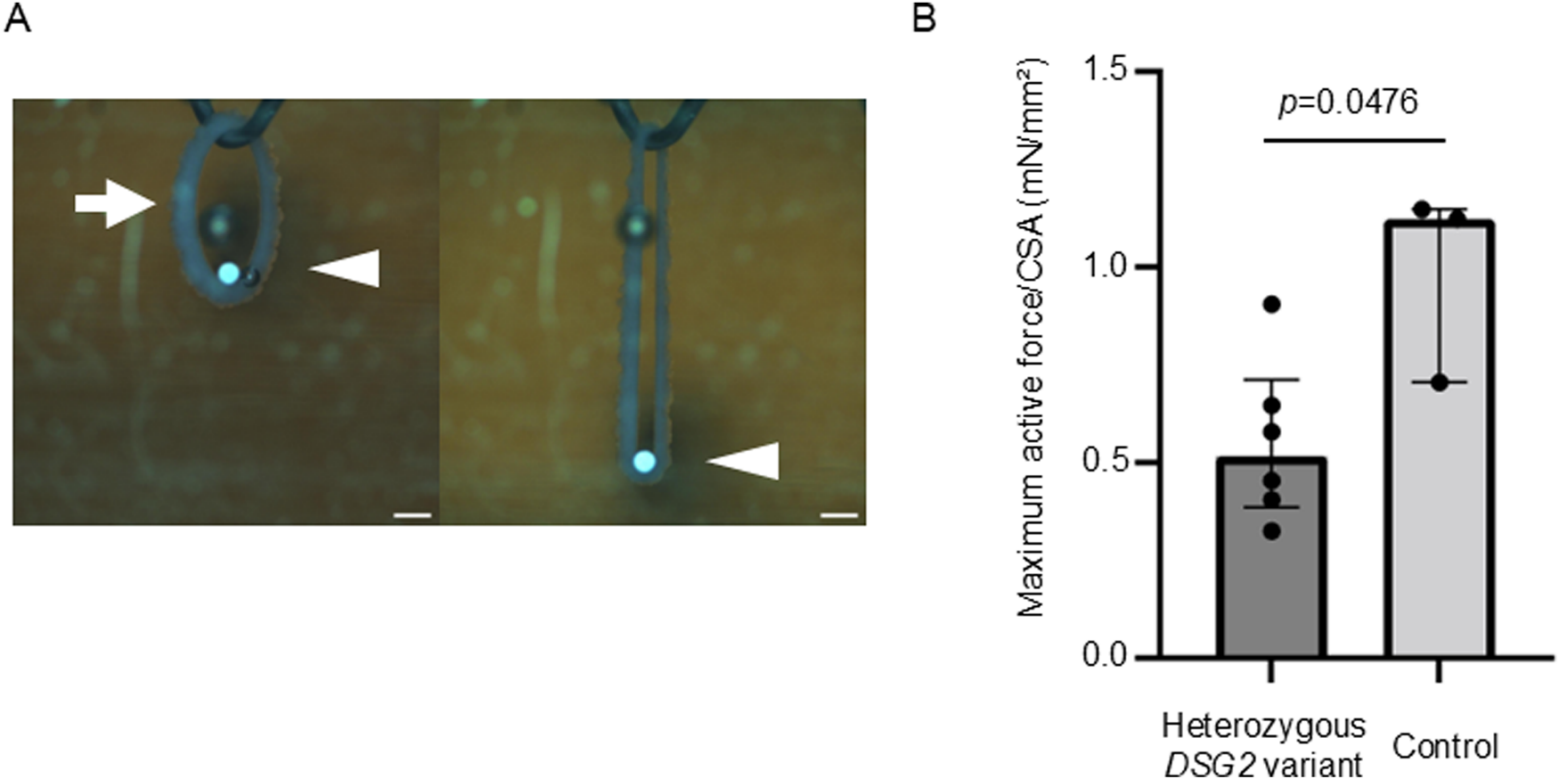
Reduced contractility in iPSC-CMs carrying a heterozygous truncating variant in *DSG2.* **A** Microforce measurement using SOTRs (arrow) compressed by a cantilever beam (arrowhead). Scale bar: 600 μm. **B** Maximum active force corrected by cross-sectional area (CSA) was compared between SOTRs generated from iPSC-CMs with a heterozygous *DSG2* variant (*n* = 6) and SOTRs generated from control iPSC-CMs (*n* = 3). The data were analyzed via the Mann‒Whitney test. Data are expressed as medians with the 25th and 75th percentiles. Statistical significance was set at *p* < 0.05.

To evaluate whether the reduced expression of desmoglein-2 due to a heterozygous truncating variant in *DSG2* potentially affects contractility in human cardiomyocytes, we generated self-organized tissue rings (SOTRs)^15^ from iPSC-CMs with or without a heterozygous frameshift variant in *DSG2* (details in “Materials and Methods”) and measured the active force as previously described^6^. A microforce test on the generated SOTRs demonstrated that the active force was significantly lower in SOTRs with the heterozygous truncating variant in *DSG2* than in control SOTRs (Fig. 5A, B), suggesting the potential pathological role of this variant in human cardiomyocytes.

## Discussion

Here, we identified four unrelated cardiomyopathy patients carrying heterozygous *DSG2* p.Arg119Ter variants who exhibited various clinical courses. The allele frequency of the *DSG2* p.Arg119Ter variant among 808 patients with nonischemic cardiomyopathy, including one homozygous patient and four heterozygous patients, was 0.0037, which was more than 50-fold greater than that reported in the general Japanese population (0.0000064)^18^. Because patients diagnosed with nonischemic cardiomyopathy and eligible for genetic analysis in our hospital require detailed cardiovascular examinations due to disease progression, the *DSG2* p.Arg119Ter variant might be more common in patients with advanced heart failure than in the general population.

Compound heterozygous *DSG2* p.Arg119Ter and p.Arg292Cys variants were identified in Pt-1. The homozygous *DSG2* p.Arg292Cys variant was identified in a patient with juvenile ARVC and sudden death^24^, and compound heterozygous *DSG2* p.Arg292Cys and p.Ser194Leu variants were identified in an ARVC patient^25^. Genetic studies of 99 unrelated Japanese ARVC probands revealed three homozygous and 11 heterozygous *DSG2* p.Arg292Cys variants in 14 of 75 Japanese ARVC patients, and their family members rarely developed ARVC-related symptoms^26^. These data suggest that the compound heterozygous *DSG2* p.Arg119Ter and p.Arg292Cys variants are the most likely cause of ARVC in Pt-1.

No cardiomyopathy-related pathological variants other than *DSG2* p.Arg119Ter were identified in Pt-2. A cohort study following 174 patients who underwent VSD closure surgery for 40 years postoperatively demonstrated that LV systolic function was impaired but stable in 21% of patients and that LVDd/Ds and fractional shortening (FS) evaluated by echocardiography were 50 ± 6/33 ± 6 mm and 35 ± 8%, respectively, 32–44 years after the operation^27^. As ventricular interactions are mediated by mechanical coupling through the ventricular septum and common myocardial fibers, inadequate deformation of the ventricular septal fibers associated with left ventricular spherization contributes to reduced contractile performance^28^. Pt-2 exhibited progression of LV dysfunction (LVDd/Ds: 64/51 mm, EF: 43%, FS: 19%) at 52 years of age compared with the previously reported natural history, suggesting that interventricular plate dysfunction combined with the *DSG2* p.Arg119Ter variant might contribute to the pathogenesis of DCM.

A novel frameshift variant, *BAG3* p.His166SerfsTer6, was identified in Pt-3. The *BAG3* p.His166SerfsTer6 mutation was considered the causative variant of DCM because the neighboring frameshift variant *BAG3* p.Pro163GlnfsTer48 is defined as pathogenic^17^, and heterozygous frameshift or stop-gain variants of *BAG3* have been identified previously in patients with DCM^29,30^. A cohort study comprising 129 individuals with *BAG3* variants demonstrated that disease penetrance in individuals over 40 years of age was 80% and that the LVEF and LVDd were 34.1 ± 13.0% and 64.5 ± 7.9 mm, respectively, at the first evaluation in patients diagnosed with DCM^21^. Echocardiographic evaluation of Pt-3 revealed more advanced LV dysfunction (LVEF: 15%, LVDd: 83 mm) than previously reported parameters, suggesting that digenic heterozygous *DSG2* p.Arg119Ter and *BAG3* p.His166SerfsTer6 variants might contribute to disease progression in Pt-3.

No cardiomyopathy-related pathological variants other than the *DSG2* p.Arg119Ter variant were identified in Pt-4, although Pt-4 was diagnosed with HCM. As our genetic analysis was based on whole-exome sequencing, we cannot exclude the possibility of noncoding intronic and intergenic variants that are associated with known HCM-causing genetic variants^31^. A cohort study evaluating genotype‒phenotype correlations in 1000 patients with HCM revealed 27 deleterious rare desmosomal variants, including *DSG2* p.Arg119Ter, in 24 (2.4%) patients with HCM and demonstrated that deleterious rare desmosomal variants are associated with distinctive clinical features, including a higher incidence of RV involvement, ventricular arrhythmias, and conduction block^32^. In HCM pathogenesis, the activation of calcium sensitivity and stress-responsive molecular pathways mediate the reprogramming of cardiac hypertrophy^22^, thereby causing continuous stress on cardiomyocytes. Therefore, the continuous myocardial stress caused by HCM pathology combined with the *DSG2* p.Arg119Ter variant may have contributed to the transition to end-stage HCM in Pt-4.

Our histopathological analysis of myocardial samples of patients demonstrated reduced reactivity for desmoglein-2 and desmoplakin in Pt-1 and Pt-3 and abnormal deposition of desmosome proteins in Pt-2. These data are consistent with a previous finding that the signal for desmoplakin as evaluated by immunohistochemical analysis was absent in the explanted heart of an ARVC patient carrying a null allele in desmoglein-2, possibly due to its abnormal redistribution into nonjunctional pools^33^. BAG3 is a cochaperone in the protein degradation machinery and has a role related to cell-to-cell junctions; in addition, *BAG3* knockdown decreases the levels of connexin 43 by changing the stability of the protein^34^. These mechanisms may underlie the decreased expression of desmoglein-2 in the heart tissue of Pt-3. Transmission electron microscopy (TEM) further revealed abnormal pale junctions and widened gaps in the intercalated discs in these myocardial samples. The abnormal redistribution of desmosome proteins due to desmoglein-2 deficiency despite preserved protein expression may cause the abnormal findings observed via electron microscopy in Pt-2. These data suggest that the structural abnormalities in intercalated discs commonly observed in patients with ARVC^23^ may contribute to the mechanism of heart failure progression in patients diagnosed with DCM. In Pt-1, fragmented and aggregated desmosomes, which are uncommon histopathological features of ARVC, were observed. Similar findings were detected in a patient with biventricular heart failure who was homozygous for the *DSG2* p.Arg119Ter variant^11^ and may be a histopathological characteristic caused by complete deficiency of desmoglein-2.

Genotypic analysis of 99 patients with ARVC revealed that *DSG2* was the most frequently affected gene in the Japanese population^26^. Combined with previous findings, our data highlight the importance of genetic screening for desmosomal gene variants in patients with broad cardiomyopathy with progressive heart failure.

## Supplementary information

Supplemantary_material
