# Supplementary material for: Four cardiomyopathy patients with a heterozygous DSG2 p.Arg119Ter variant
Source: Hum Genome Var. 2024 Dec 20;11:47. doi: 10.1038/s41439-024-00304-w (PMC11661998; doi:10.1038/s41439-024-00304-w)
Supplement: Supplementary file 1 — Supplemantary_material [file 41439_2024_304_MOESM1_ESM.pdf]

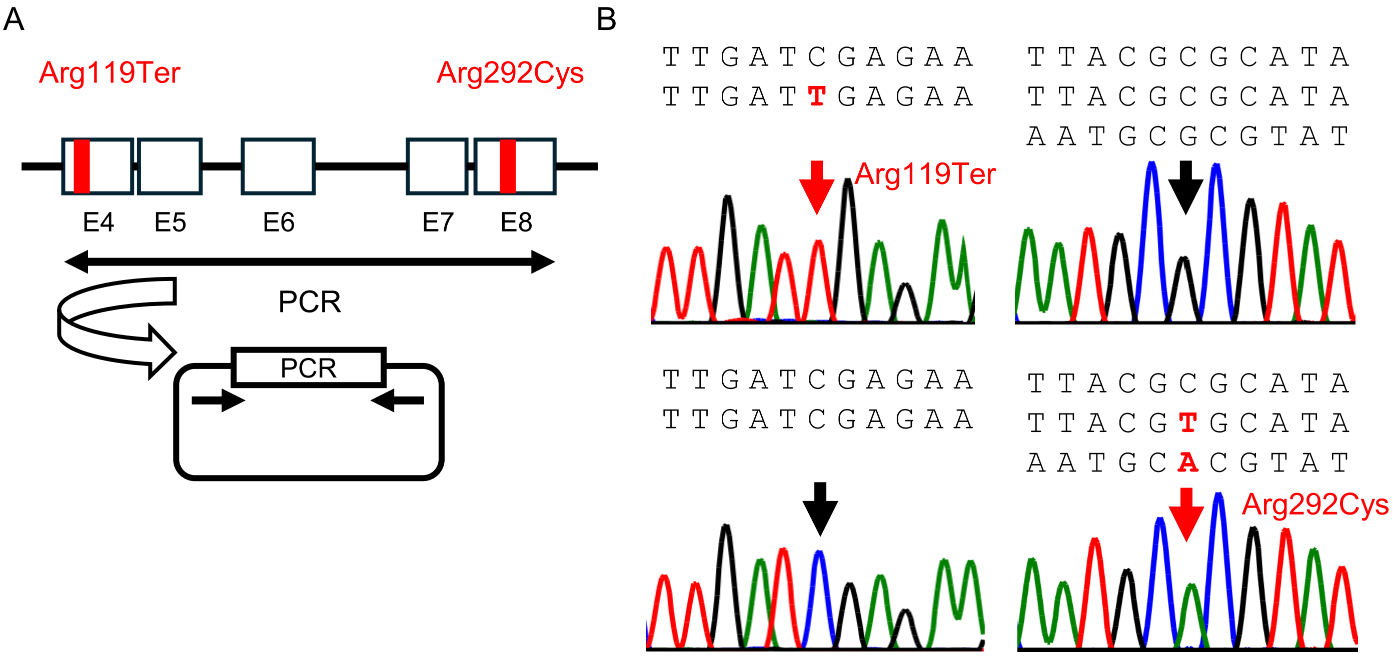

Supplementary Figure 1. Sanger sequence analyses to confirm the compound heterozygous variants in Pt-1. (A) Genomic region of *DSG2* spanning from exon 4 (E4) to exon 8 (E8) was amplified by PCR, cloned into the cloning vector, then analyzed by Sanger sequence using the bidirectional PCR primers (arrows). (B) 355C>T (Arg119Ter) variant and wild type (WT) sequence were located on the same allele (upper), while WT sequence and 874C>T (Arg292Cys) variant were located on the same allele (lower). The identified variants are highlighted in red. Red and black arrows indicate the identified variants and WT sequences, respectively.

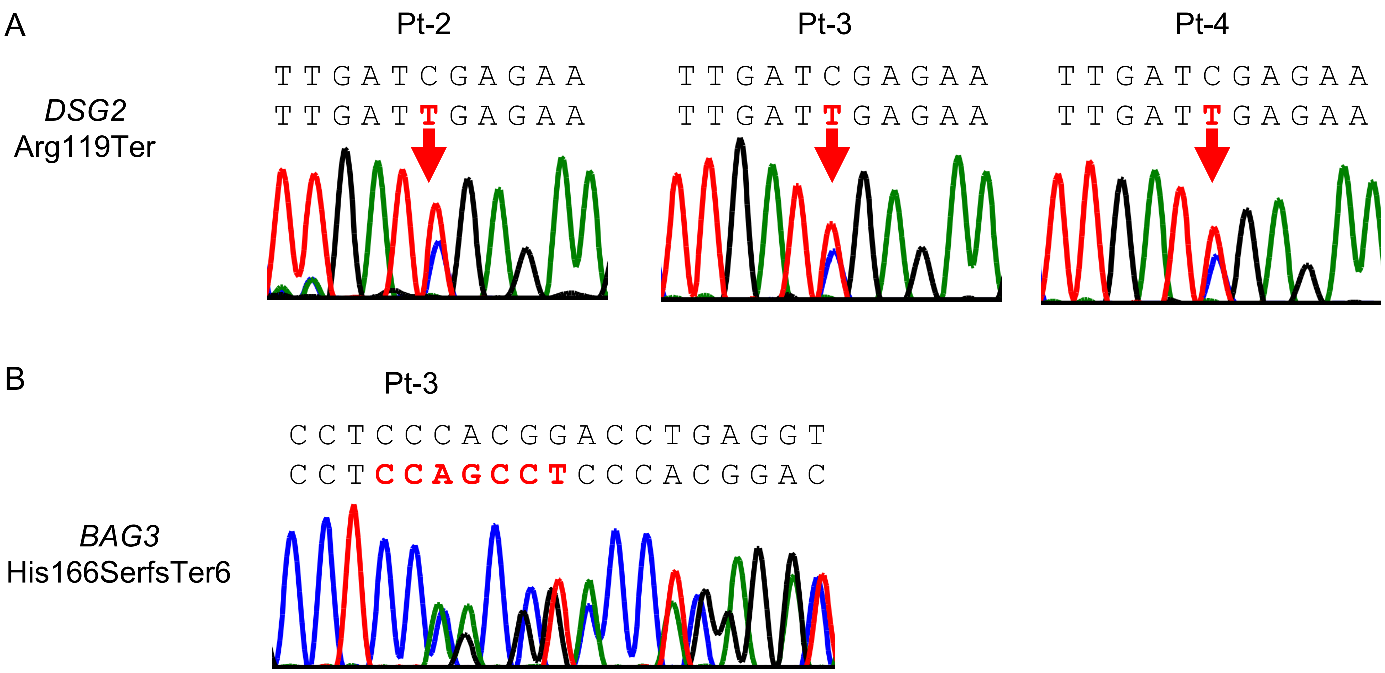

Supplementary Figure 2. Sanger sequencing results of Pt-2, Pt-3, and Pt-4.

Heterozygous *DSG2* (c.355C>T, p.Arg119Ter) variant in Pt-2, Pt-3, and Pt-4 (upper) and heterozygous *BAG3* (c.486_487insCCAGCCT, p.His166SerfsTer6) variant in Pt-3 (lower) were confirmed by direct Sanger sequencing analysis. The identified variants are highlighted in red. Red arrows indicate the location of heterozygous variants.

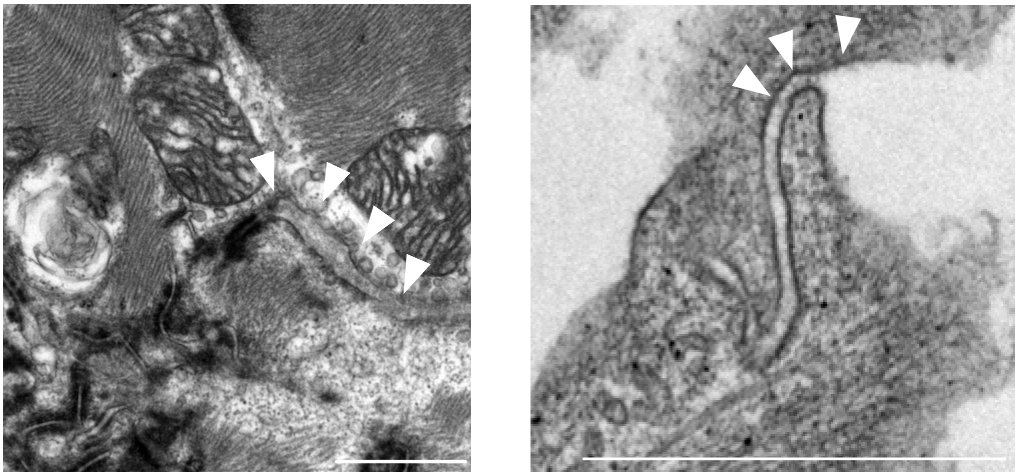

Supplementary Figure 3. Transmission electron microscope images of the LV myocardium obtained from the patient carrying the homozygous *DSG2* p.Arg119Ter variant (left) and the cardiomyocytes differentiated from the patient-derived iPS cells (right).

Disrupted desmosomes (arrowheads) were observed. Scale bar: 1 μm.

Supplementary Table 1. Primers for Sanger sequence analysis.

Oligo DNA	sequence (5'-3')
*DSG2*_R119X-R292C_F	AGGGATTACAGAGCCACCTTTTGGT
*DSG2*_R119X-R292C_R	CAACTTGGCCCACGCCAGTT
*DSG2*_R119X_F	ACTGCCATTCCCCTAGTCCAAGTCA
*DSG2*_R119X_R	GCATCCAAAGCGTAACCTGTTAGC
*BAG3*_H166Sfs_F	TTTCTAACCAGCCTGTGTTTCTCC
*BAG3*_H166Sfs_R	CTGAAGAACAGCCCTATGAGATGC

Supplementary Table 2. Pathogenic genetic variants identified in the four patients with *DSG2* (c.C355T, p.R119X).

Patient	Diagnosis	Chromosome	Position (hg38)	Ref	Alt	Gene	Zygosity	Base change	Amino acid change	Variant type	jMorp frequency	gnomAD frequency	HGMD class	ClinVar significance
Pt-1	ARVC	chr18	31520941	C	T	*DSG2*	Heterozygous	c.355C>T	p.Arg119Ter	stopgain	0.000065	0.00001207	DM?	Conflicting interpretations of pathogenicity
		chr18	31524748	C	T	*DSG2*	Heterozygous	c.874C>T	p.Arg292Cys	nonsynonymous SNV	0.002621	0.00004812	DM?	Conflicting interpretations of pathogenicity
		chr6	133462710	C	A	*EYA4*	Heterozygous	c.508C>A	p.Pro170Thr	nonsynonymous SNV	.	.	.	.
		chr2	178548031	C	T	*TTN*	Heterozygous	c.66400G>A	p.Glu22134Lys	nonsynonymous SNV	.	.	.	.
		chr10	74082542	C	A	*VCL*	Heterozygous	c.872C>A	p.Pro291Gln	nonsynonymous SNV	.	.	.	.
Pt-2	DCM post VSD closure	chr18	31520941	C	T	*DSG2*	Heterozygous	c.355C>T	p.Arg119Ter	stopgain	0.000065	0.00001207	DM?	Conflicting interpretations of pathogenicity
Pt-3	DCM	chr18	31520941	C	T	*DSG2*	Heterozygous	c.355C>T	p.Arg119Ter	stopgain	0.000065	0.00001207	DM?	Conflicting interpretations of pathogenicity
		chr10	119670156	-	CCAGCCT	*BAG3*	Heterozygous	c.486_487ins CCAGCCT	p.His166Ser fsTer6	frameshift insertion	.	.	.	.
Pt-4	End-stage HCM	chr18	31520941	C	T	*DSG2*	Heterozygous	c.355C>T	p.Arg119Ter	stopgain	0.000065	0.00001207	DM?	Conflicting interpretations of pathogenicity
		chr1	237614556	G	C	*RYR2*	Heterozygous	c.5428G>C	p.Val1810Leu	nonsynonymous SNV	0.00195	0.00008441	DM	Conflicting interpretations of pathogenicity
		chr2	178548412	G	A	*TTN*	Heterozygous	c.66019C>T	p.Arg22007Cys	nonsynonymous SNV	0.000271	0.00005238	DM?	Conflicting interpretations of pathogenicity

DM: disease-causing mutation, DM?: likely disease-causing mutation
